# Purtscher’s retinopathy in scleroderma

**DOI:** 10.3205/oc000096

**Published:** 2019-03-01

**Authors:** Mazen Alzahrani, Muhammad Abdul Rehman, Thamer Basodan, Islam Adnan, Muhammad Akhtar

**Affiliations:** 1 Department of Ophthalmology, East Jeddah Hospital, Jeddah, Saudi Arabia; 2 Department of Internal Medicine, East Jeddah Hospital, Jeddah, Saudi Arabia

**Keywords:** scleroderma, Purtscher’s retinopathy, hypertension retinopathy, renal disease, hypertension

## Abstract

We report a unique case of a 37-year-old patient, a known case of scleroderma, complaining of bilateral acute visual disturbance, which was diagnosed later on as Purtscher’s retinopathy. After systemic assessment, she was diagnosed with bilateral kidney disease, consequently requiring further management accordingly.

## Introduction

Systemic sclerosis is a rare, autoimmune, gradually progressive chronic disease affecting the immune system, blood vessels, and connective tissue in around 2.5 million people worldwide. The immune system produces connective tissue and attacks normal tissue in the entire body. The increase of collagenous material can cause scarring and affect skin, joints, tendons, heart, oesophagus, blood vessels, kidneys, blood pressure, and the gastric system, gradually impairing their functionality and in some cases leading to potentially serious complications and death [1]. Abnormalities in retinal vessels may indicate other body changes elsewhere, suggesting systemic pathology [2].

We reported here Purtscher’s retinopathy, which is a rare finding, in a patient whose hypertensive retinopathy suggested an underlying renal organ disease, secondary to scleroderma. We conducted this study alongside a clinical profile of the scleroderma patient.

## Case description

A woman of thirty-seven years was diagnosed with scleroderma 2 weeks previously and presented to the Emergency Department of East Jeddah Hospital. She was complaining of a frontal and occipital headache and accompanying vomiting and nausea for the 4 days prior, associated with visual disturbances in the form of black spots, decreased and blurred vision. She had no history of loss of consciousness, fits, and odynophagia or dysphagia. The patient had a past history of progressive thickness and tightness in face, chest, and hands, with associated hypo-pigmented limb skin, dry mouth with stiffness experienced on closing the mouth, and joint pain for one year. The rest of her systemic review was unremarkable. On examination, blood pressure (BP) was 215/110. The patient was conscious, oriented and alert, she looked pale with no jaundice or lymphadenopathy. Her hands looked red, she had palmar telangiectasia and tight thickened skin without deformity. The patient had intact joint movement and Raynaud phenomenon was positive for both fingers. The rest of the examination was irrelevant.

**Ocular history: Pertinent findings – clinical**

**Figure 1:**
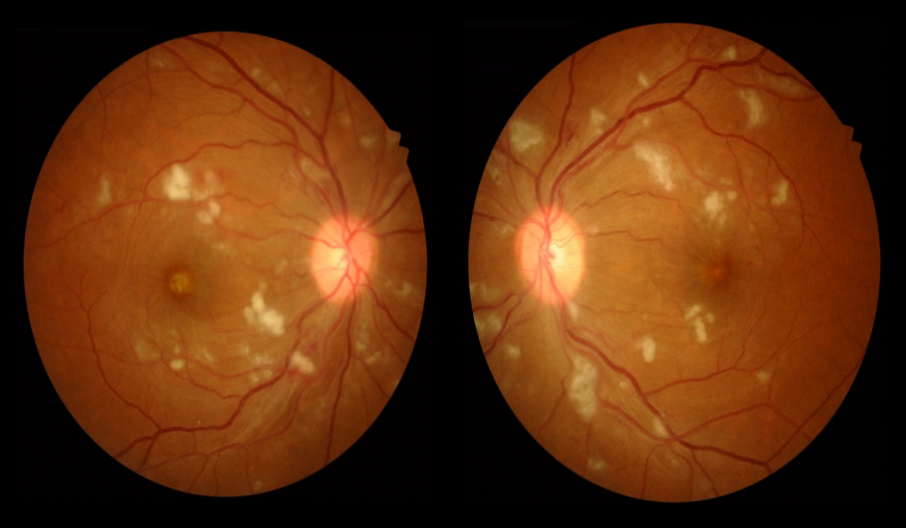
Fundus pictures: bilateral multiple cotton wool exudates, dot hemorrhage known as Purtscher’s flecken, macula star pattern cup to disc ratio 0.31

- Visual acuity: OD 20/200, OS 20/80
- Eye movement: fully intact, she had no nystagmus or abnormality of cranial nerves
- Slit lamp: lids/lashes, conjunctiva and cornea were normal
- Goldman tonometry: OD 18 mmHg, OS 16 mmHg at 11:30 AM
- Fundus exam (Figure 1 (Fig. 1)): multiple exudate and cotton wool spots with dot haemorrhage known as Purtscher’s flecken
- Macula: macula star pattern with pigmented epithelium detached. Cup to disc ratio: 0.31

**Figure 2:**
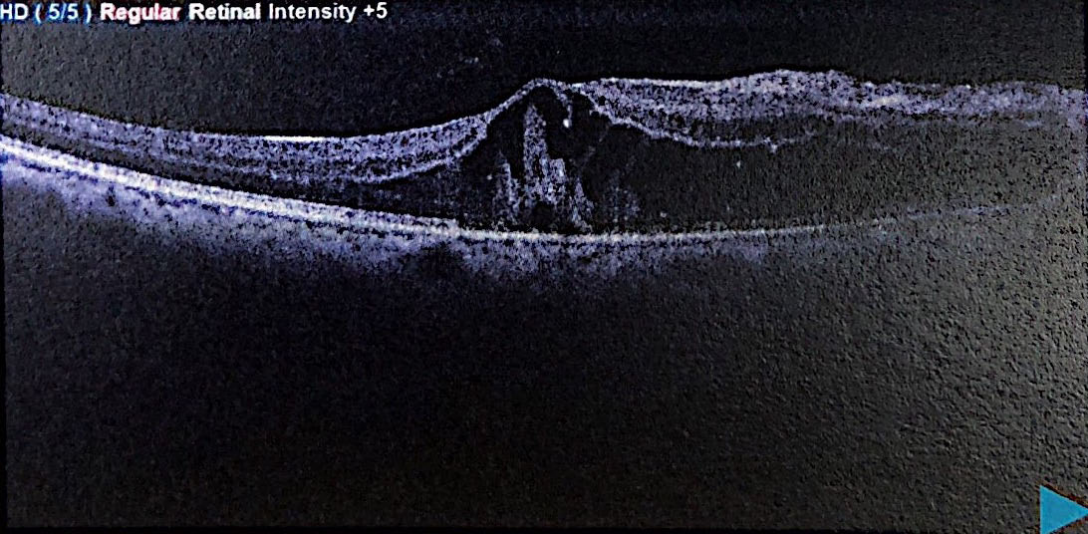
Right OCT: bilateral foveal serous detachment with CME and macular retinoschisis

**Figure 3:**
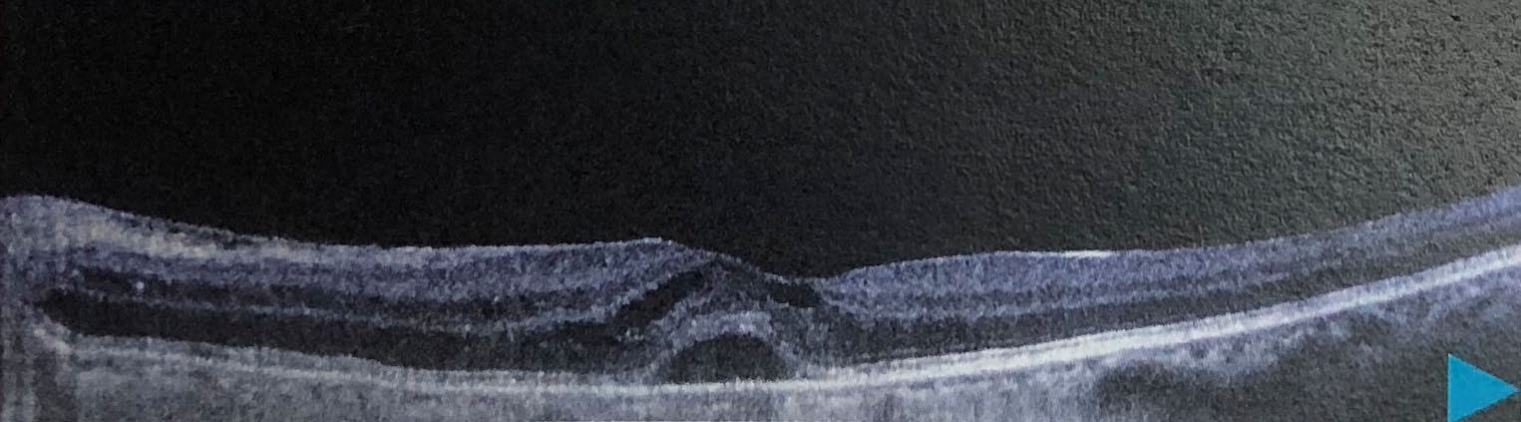
Left OCT: separation in the inner retinal layers with a variable degree of macular oedema

**Figure 4:**
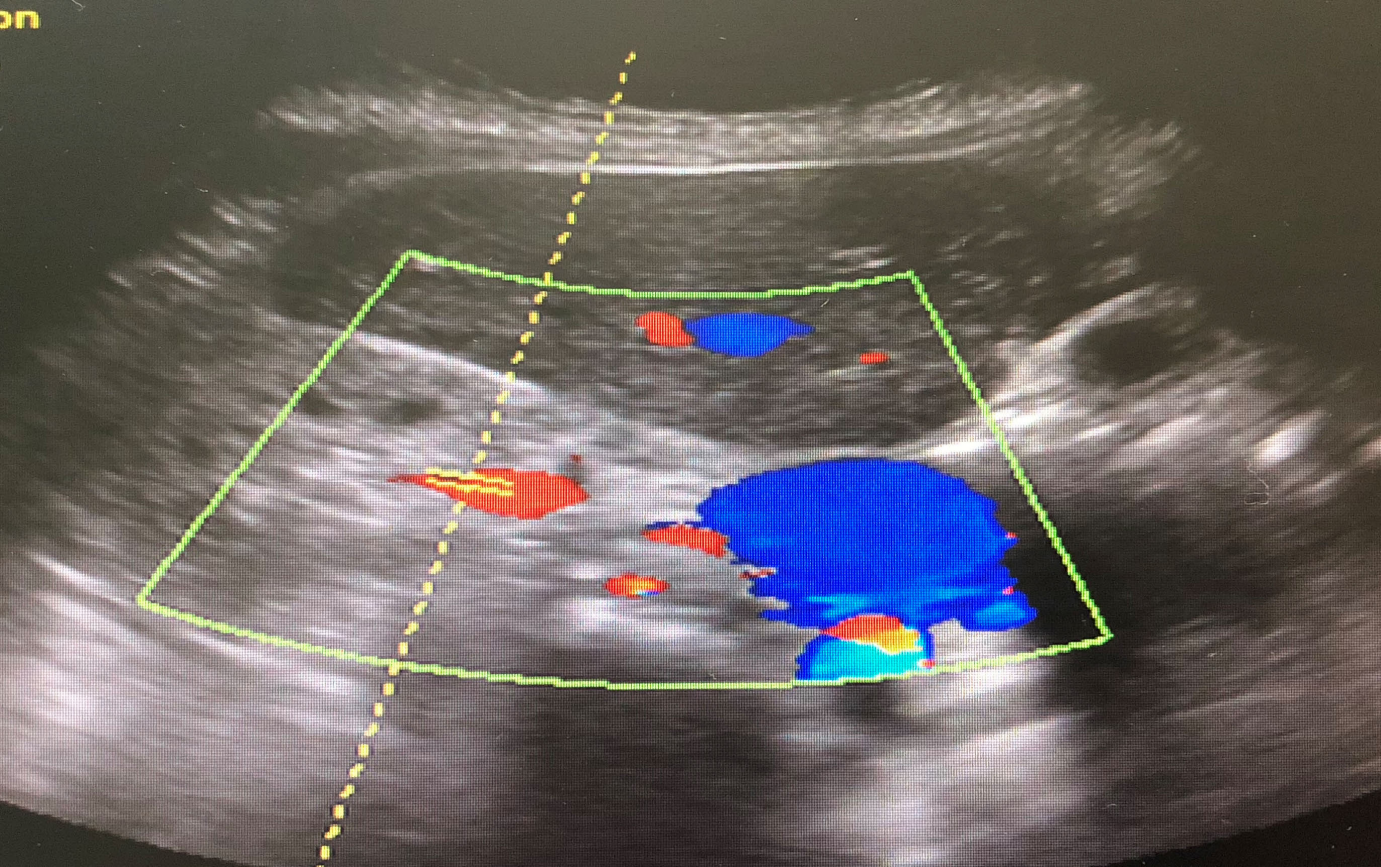
Renal Doppler ultrasound: abnormal resistive index bilaterally with abnormally echogenic kidney, suggestive of medical renal disease

The patient was admitted, laboratory results obtained are shown in Table 1 (Tab. 1). Optical coherence tomography (OCT) (Figure 2 (Fig. 2) and Figure 3 (Fig. 3)) displayed bilateral foveal serous detachment with CME and macular retinoschisis, separation in the inner retinal layers with a variable degree of macular oedema for the right eye and left eye, respectively. Kidney ultrasound indicated renal disease (Figure 4 (Fig. 4)). The other laboratory results were within normal range. We did not request fundus florescence angiography due to the patient’s renal status. Later on, diagnosis of scleroderma with renal crisis was made. After receiving extensive anti-hypertensive medication following internist and ICU physician attention, the patient’s BP returned to 140/90, and she was transferred from the ICU for a multidisciplinary approach.

## Discussion

Purtscher’s retinopathy stays controversial and mirrors different etiologies such as acute pancreatitis [3], renal disease, and connective tissue disorders [4]. It causes retinal haemorrhage and retinal whitening, with ischaemic infarctions (cotton wool spots) with or without haemorrhages (dot and blot), and optic disc oedema associated with a decrease in visual acuity. The Purtscher flecken manifest as areas of inner retinal whitening with spot areas with a finly drawn line around 50 µm between the affected retinal area and the normal vessels. In our case, we found typical fundus appearance of Purtscher flecken (Figure 1 (Fig. 1)) and it fulfilled the Agrawal et al. criteria, with the spot confined to the posterior pole, no history of trauma, no vessels embolus, and with retinal blot or dot. Our case also had renal failure, one of a variety of potential diseases ([5], Table 2 (Tab. 2)). The aggravator in our case is dual, by vessel microangiopathy of scleroderma and secondary hypertension to the renal abnormality. It is caused either by being attacked by collagen formation in the form of deposits on the capillary endothelium wall by scleroderma autoimmune disease process or ischaemia leading to arteriole blockage [6].

The eye complications of scleroderma are varied and include a decrease of eye tearing, keratitis and keratoconjunctivitis sicca present in up to 70% of cases [7]. The retinal changes can be found along with other vasculitis connective tissue disorders, such as systemic lupus erythematosus and not necessary due to a rise in systemic hypertension; one case was reported with retinal changes without a rise in blood pressure [8].

## Conclusion

Our case has shown a Purtscher’s retinopathy in a scleroderma patient, complicated by a hypertensive renal crisis.

Early diagnosis, control of scleroderma, and routine eye exams are necessary and would suppress a variety of organ complications. Early collaboration with an ophthalmologist is favorable to reduce morbidity and mortality.

## Notes

### Competing interests

The authors declare that they have no competing interests.

### Informed consent

Informed consent was obtained by the patient regarding the publication of the case report. 

## Figures and Tables

**Table 1:**
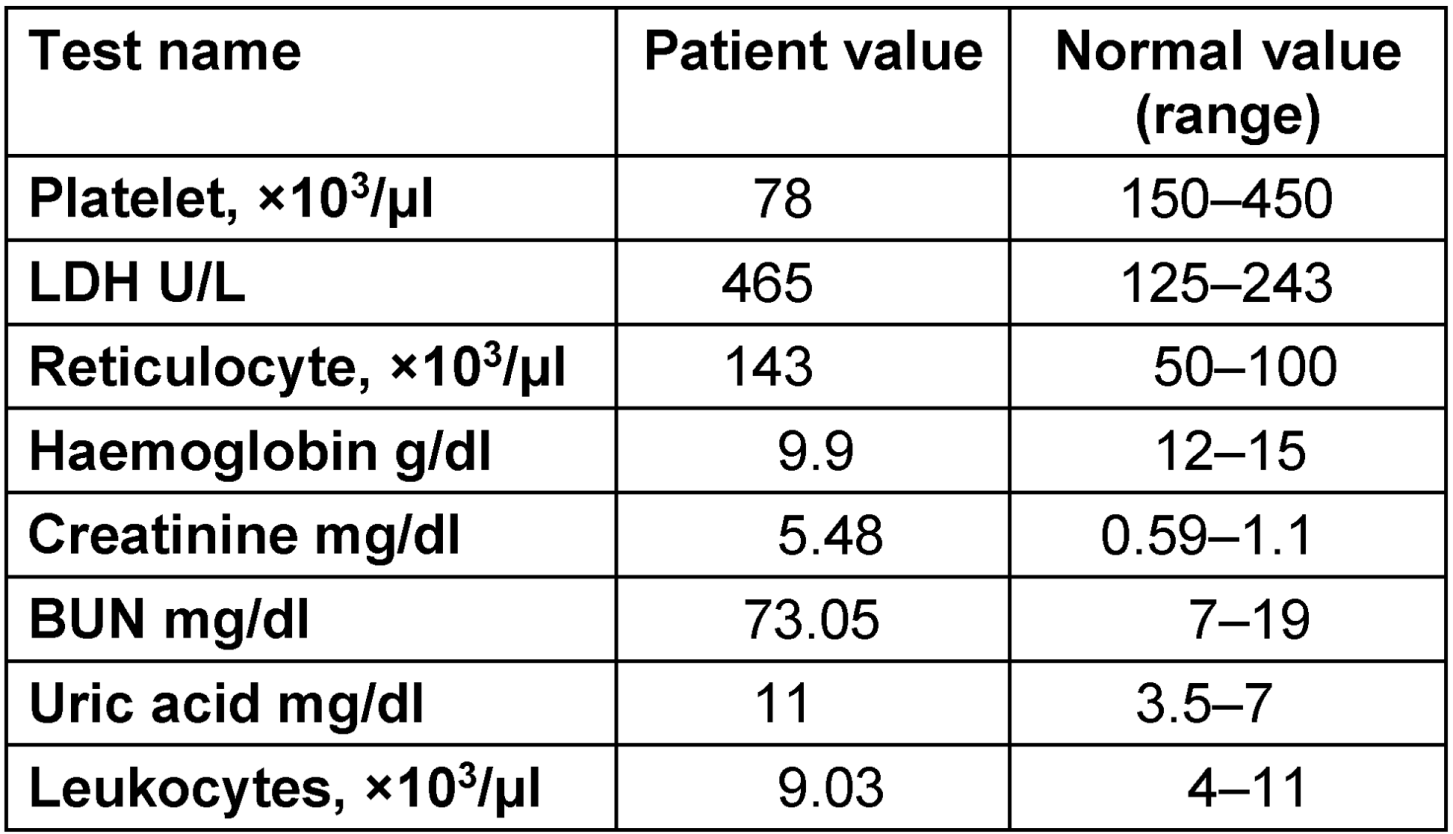
Laboratory test results

**Table 2:**
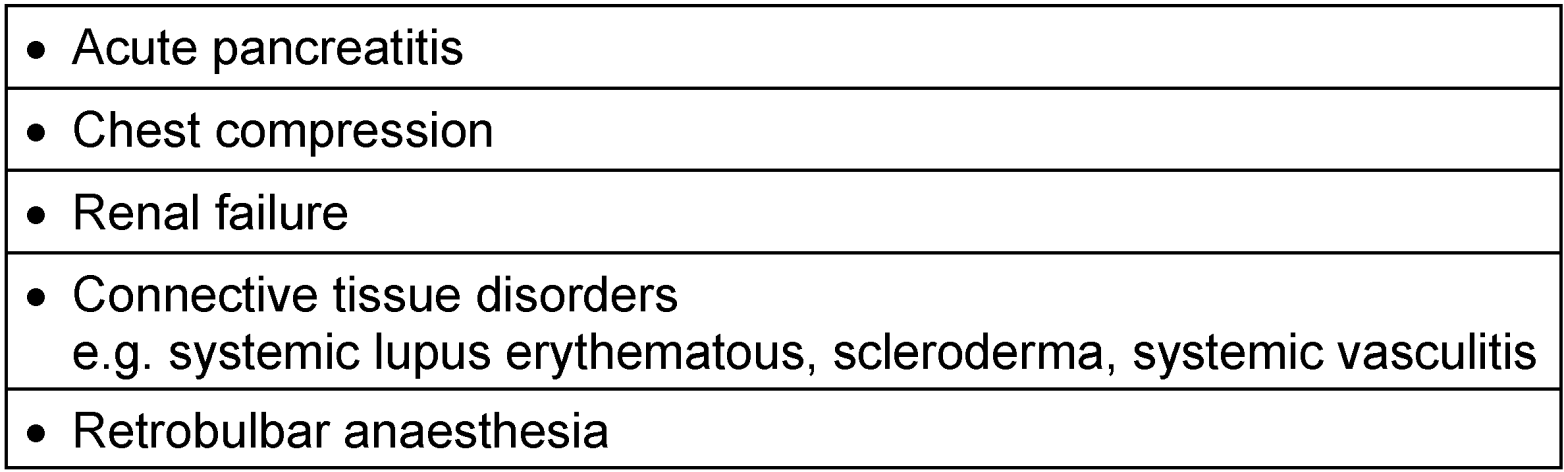
Known diseases or events with Purtscher’s retinopathy [9]
